# Diel Protein Regulation of Marine Picoplanktonic Communities Assessed by Metaproteomics

**DOI:** 10.3390/microorganisms9122621

**Published:** 2021-12-18

**Authors:** Augustin Géron, Johannes Werner, Philippe Lebaron, Ruddy Wattiez, Sabine Matallana-Surget

**Affiliations:** 1 Proteomics and Microbiology Department, University of Mons, 7000 Mons, Belgium; augustin.geron@umons.ac.be (A.G.); ruddy.wattiez@umons.ac.be (R.W.); 2 Division of Biological and Environmental Sciences (BES), Faculty of Natural Sciences, University of Stirling, Stirling FK9 4LA, UK; 3 High Performance and Cloud Computing Group, Zentrum für Datenverarbeitung (ZDV), Eberhard Karls University of Tübingen, 72074 Tübingen, Germany; johannes.werner@uni-tuebingen.de; 4 Department of Biological Oceanography, Leibniz Institute for Baltic Sea Research, 18119 Rostock, Germany; 5 USR3579, Le Laboratoire de Biodiversité et Biotechnologies Microbiennes (LBBM) de l’Observatoire Océanologique, UPMC University Paris 06, Sorbonne Universités, 66651 Banyuls-sur-Mer, France; lebaron@obs-banyuls.fr

**Keywords:** diel cycle, picoplankton, microbial communities, metaproteomics

## Abstract

The diel cycle is of enormous biological importance in that it imposes temporal structure on ecosystem productivity. In the world’s oceans, microorganisms form complex communities that carry out about half of photosynthesis and the bulk of life-sustaining nutrient cycling. How the functioning of microbial communities is impacted by day and night periods in surface seawater remains to be elucidated. In this study, we compared the day and night metaproteomes of the free-living and the particle-attached bacterial fractions from picoplanktonic communities sampled from the northwest Mediterranean Sea surface. Our results showed similar taxonomic distribution of free-living and particle-attached bacterial populations, with *Alphaproteobacteria*, *Gammaproteobacteria* and *Cyanobacteria* being the most active members. Comparison of the day and night metaproteomes revealed that free-living and particle-attached bacteria were more active during the day and the night, respectively. Interestingly, protein diel variations were observed in the photoautotroph *Synechococcales* and in (photo)-heterotrophic bacteria such as *Flavobacteriales, Pelagibacterales* and *Rhodobacterales*. Moreover, our data demonstrated that diel cycle impacts light-dependent processes such as photosynthesis and UV-stress response in *Synechococcales* and *Rhodobacterales*, respectively, while the protein regulation from the ubiquitous *Pelagibacterales* remained stable over time. This study unravels, for the first time, the diel variation in the protein expression of major free-living and particle-attached microbial players at the sea surface, totaling an analysis of eight metaproteomes.

## 1. Introduction

Microorganisms in marine ecosystems are extremely diverse, dominate biomass and play key roles in biogeochemical processes [1,2]. Picoplankton (i.e., the microorganisms of a size ranging between 0.2–2 µm) carry out up to half of the world ocean’s primary production and the bulk of life-sustaining nutrient cycling [3]. Marine picoplanktonic communities are composed of both free-living and particle-attached microorganisms. A comparison of these bacterial fractions in coastal environments showed differences in cell abundance [4], morphology and metabolic activity [5]. In terms of phylogenetic diversity, studies suggested that free-living and particle-attached communities were fundamentally different [6,7,8], while others reported high similarities between both fractions [9,10,11]. 

The diel oscillation of solar radiation reaching the Earth’s surface temporally structures biological events, activities, and physiological processes across all kingdoms of life [12]. Day/night changes were found to modulate the functioning of sea surface picoplanktonic communities on the following processes: metabolites consumption [13,14], viral infection [15], DNA/protein synthesis and dissolved organic carbon distribution [16]. In the northwest (NW) Mediterranean Sea, under oligotrophic conditions, free-living bacteria were found to be more abundant than particle-attached bacteria [11]. Moreover, bacterial activity estimated from ^3^H-leucine incorporation rates showed that free-living bacteria contributed the most to bacterial activity during the day and night, while higher cell-specific activity was found in particle-attached bacteria [11].

The development of omics approaches has improved the understanding of marine microbial assemblages [17]. Environmental metatranscriptomic studies reported distinct day and night metabolic activities of marine microorganisms from oligotrophic marine environments [18,19]. Microbial assemblages isolated from the North Pacific subtropical gyre showed an overabundance of transcripts for photosynthesis, C1 metabolism and oxidative phosphorylation during the day [18]. However, housekeeping activities, such as amino acid or vitamin biosynthesis, were overrepresented at night. Transcripts of genes involved in light-driven processes were found in higher abundance during daytime, in surface marine picoplankton sampled from the Western English Channel [19]. Diel transcriptional rhythms in *Cyanobacteria* were evidenced together with diel oscillations in different heterotrophic bacterial groups including photoheterotrophic and proteorhodopsin-containing bacteria [20]. Phylogenetic analysis of gene transcripts revealed that the composition of marine microbial assemblages was stable over the day and night periods, especially for the most abundant taxa [20]. To what extent picoplanktonic communities are collectively entrained by the day and night periods and rhythmically regulate their protein expression remains poorly documented.

Metaproteomics allows for the characterization of the total proteins within microbial communities [21] and, in association with other omics, deciphers the functional complexity of microbial ecosystems [22]. Since the first environmental metaproteomic study [23], this method rapidly expanded and broadened our knowledge of marine ecosystems [24]. For example, marine metaproteomic revealed the extreme microbial competition for nutrients in oligotrophic systems [25,26], provided insights into the dynamics in organic matter transformation by microorganisms [27,28] and showed the spatiotemporal variation in metabolic activities in oceanic plankton communities [29]. Environmental metaproteomics is a growing discipline, hampered by the inherent complexity of natural microbial assemblages [30]. Over the past years, the development of sampling protocols, fast scanning high-resolution mass spectrometers and protein identification and annotation software significantly improved the metaproteomic workflow [17,24,31]. Marine oligotrophic waters still present significant challenges for metaproteomic studies because of (i) the low bacterial biomass preventing high protein rate recovery, (ii) the difficulty of separating prokaryotes from microeukaryotes and (iii) the protein inference issue [17,24,30].

In this study, we compared the day and night metaproteomes of both free-living (0.2–0.8 µm) and the particle-attached (>0.8 µm) bacterial fractions sampled at the surface of NW Mediterranean Sea in summer. A combined protein search database allowed us to maximize the number of protein identifications [30]. The protein inference issue, commonly encountered in metaproteomics, was overcome using taxonomic and functional consensus protein annotation, providing an accurate assessment of the diel variation [32]. To the best of our knowledge, this is the first metaproteomics study that depicts day and night metaproteomes of marine picoplankton.

## 2. Materials and Methods

### 2.1. Water Sampling

Seawater sampling was performed in summer (June 2014) at the SOLA station, located 500 m offshore of Banyuls-sur-mer, in the NW Mediterranean Sea (42°49′ N, 3°15′ W). Samples were collected at sunset and sunrise during two consecutive days and consisted of 70 L of sea surface water each. Water was pre-filtered onto a 5 µm mesh and sequentially filtered through 0.8 and 0.2 µm pore-sized filters (polyethersulfone membrane filters, PES, 142 mm, Millipore, Burlington, Massachusetts, United States) to collect the particle-attached and the free-living bacterial fractions, respectively. A pre-filtration onto a 5 µm mesh was mandatory to prevent the studied bacterial fractions (0.8 µm and 0.2 µm) from being contaminated by eukaryotic organisms, which would otherwise alter the metaproteomic workflow. As a reminder, metaproteomics allows for the characterization of the most abundant proteins. The eight filters were flash frozen in liquid nitrogen before storage at −80 °C.

The physicochemical parameters were provided by the Service d’Observation en Milieu Littoral (SOMLIT). On site average temperature and salinity in June were 18.7 ± 0.7 °C and 37.8 ± 0.1 psu, respectively, as provided by SOMLIT. pH was stable, with an average of 8.26 ± 0.04. Nutrient concentrations averaged 0.03 ± 0.01 µM NH_4_^+^, 0.05 ± 0.03 µM NO_3_^−^, 0.01 ± 0.001 µM NO_2_^−^, 0.02 ± 0.01 µM PO_4_^3−^ and 0.75 ± 0.09 µM Si(OH)_4_.

### 2.2. Protein Isolation

The filters were cut using aseptic procedures and suspended in a lysis buffer containing 8 M Urea/2 M Thiourea, 10 mM HEPES and 10 mM dithiothreitol. Filters were subjected to five freeze–thaw cycles in liquid N_2_ to release cells from the membrane. Cells were mechanically broken by sonication on ice (5 cycles of 1 min with tubes on ice, amplitude 40%, 0.5 pulse rate) and subsequently centrifuged at 16,000 *g* at 4 °C for 15 min. To remove particles that did not pellet during the centrifugation step, the protein suspension was filtered through a 0.22 µm syringe filter and transferred into a 3 kDa cutoff Amicon Ultra-15 filter unit (Millipore) for protein concentration. Proteins were precipitated with cold acetone overnight at −80 °C, with an acetone/aqueous protein solution ratio of 4:1. Total protein concentration was determined by a Bradford assay, using the Bio-Rad Protein Assay kit (Bio-Rad, Hertfordshire, UK) according to manufacturer’s instructions, with bovine γ-globulin as a protein standard. Protein samples were reduced with 25 mM dithiothreitol (DTT) at 56 °C for 30 min and alkylated with 50 mM iodoacetamide at room temperature for 30 min. Gel-free liquid chromatography tandem mass spectrometry was performed utilizing a trypsic digestion (sequencing grade modified trypsin, Promega, Madison, Wisconsin, États-Unis) overnight at 37 °C, with an enzyme/substrate ratio of 1:25.

### 2.3. Liquid Chromatography Tandem Mass Spectrometry Analysis

Purified peptides from digested protein samples were identified using a label-free shotgun approach on an UHPLC-HRMS platform composed of an eksigent 2D liquid chromatograph and an AB SCIEX Triple TOF 5600. Peptides were separated on a 25 cm C18 column (Acclaim pepmap 100, 3 μm, Dionex, Sunnyvale, Californie, États-Unis) by a linear acetonitrile (ACN) gradient (5–35% (*v*/*v*), in 15 or 120 min) in water containing 0.1% (*v*/*v*) formic acid at a flow rate of 300 nL min^−1^. Mass spectra (MS) were acquired across 400–1500 *m*/*z* in high-resolution mode (resolution >35,000) with 500 ms accumulation time. Six microliters of each fraction were loaded onto a pre-column (C18 Trap, 300 µm i.d. × 5 mm, Dionex) using the Ultimate 3000 system, delivering a flow rate of 20 µL/min loading solvent (5% (*v*/*v*) acetonitrile (ACN), 0.025% (*v*/*v*) TFA). After a 10 min desalting step, the pre-column was switched online with the analytical column (75 µm i.d.× 15 cm PepMap C18, Dionex) equilibrated in 96% solvent A (0.1% (*v*/*v*) formic acid in HPLC-grade water) and 4% solvent B (80% (*v*/*v*) ACN, 0.1% (*v*/*v*) formic acid in HPLC-grade water). Peptides were eluted from the pre-column to the analytical column and then to the mass spectrometer, with a gradient from 4–57% solvent B for 50 min and 57–90% solvent B for 10 min at a flow rate of 0.2 µL min^−1^ delivered by the Ultimate pump. Positive ions were generated by electrospray and the instrument was operated in a data-dependent acquisition mode, described as follows: MS scan range: 300–1500 *m*/*z*, maximum accumulation time: 200 ms, ICC target: 200,000. The top 4 most intense ions in the MS scan were selected for MS/MS in dynamic exclusion mode: ultrascan, absolute threshold: 75,000, relative threshold: 1%, excluded after spectrum count: 1, exclusion duration: 0.3 min, averaged spectra: 5 and ICC target: 200,000. Metaproteomic raw data are available in the iProx public platform [33] (Project ID: IPX0002008000; Subproject IDs: IPX0002008001 (free-living fractions), IPX0002008002 (particle-attached fractions)).

### 2.4. Ocean Sampling Day 2014 Metagenomic Data Set

Metagenomic data from the Ocean Sampling Day 2014 (OSD14) were downloaded from the EMBL-EBI MGnify platform (Project number: ERP009703, sample: OSD14_2014_06_2m_NPL022, run ID: ERR771073). Briefly, water was sampled at the same location (42°49′ N, 3°15′ W) and month (June 2014) as our metaproteomic study, using a CTD rosette with Niskin bottles. Water was filtered on a 0.22 µm pore-sized filter and stored at −80 °C until subsequent DNA extraction and sequencing. Illumina sequencing was performed using an Illumina MiSeq instrument, and reads were processed using the OSD14 pipeline version 4.0. Briefly, paired-end overlapping reads were merged using SeqPrep [34] and low-quality sequences were trimmed using Trimmomatic [35]. Adapter sequences and sequences <100 nucleotides in length were removed using Biopython [36]. Infernal [37] was used for ncRNAs identification and cmsearch deoverlap script was used to remove lower scoring overlaps. Genes were called using FragGeneScan (short reads) [38] and Prodigal [39]. InterProScan [40] was used for gene identification and MAPseq for taxonomic annotation [41].

### 2.5. Databases Creation and Protein Identification

Protein identification was performed with ProteinPilot (ProteinPilot Software 5.0.1; Revision: 4895; Paragon Algorithm: 5.0.1.0.4874; AB SCIEX, Framingham, MA, USA) (Matrix Science, London, UK; v. 2.2) (File S1). Paragon searches were conducted using LC MS/MS Triple TOF 5600 System instrument settings. Other parameters used for the search were as follows: Sample Type: Identification, Cys alkylation: Iodoacetamide, Digestion: Trypsin, ID Focus: Biological Modifications and Amino acid substitutions, Search effort: Thorough ID, Detected Protein Threshold [Unused ProtScore (Conf)] > 0.05 (10.0%). 

Three protein search databases (DBs) were created with mPies v. 0.9 [32], using the OSD14 metagenome as a template. The three DBs were: (i) a non-assembled metagenome-derived DB (NAM-DB), (ii) an assembled metagenome-derived DB (AM-DB) and (iii) a taxonomy-derived DB (TAX-DB) [30]. An initial protein search was performed for each sample against the three DBs individually. Subsequently, each DB was restricted to the protein sequences identified in the first-round search. The resulting DBs were merged, and redundant protein sequences were removed, leading to a unique combined DB per sample. Finally, a second protein search was performed for each sample against their respective combined DB, except for the 0.8 µm samples, where all combined DB were merged to increase the identification yield. The identified proteins were selected based on a FDR threshold of 1%, calculated at the protein level was used for each protein searches (File S2). Proteins identified with one single peptide spectrum were validated by manual inspection of the MS/MS spectra, ensuring that a series of at least five consecutive sequence-specific b-and y-type ions was observed. 

### 2.6. Protein Annotation and Downstream Analyses

Identified proteins were annotated using mPies [32]. The mPies tool used Diamonds [42] to align each identified protein sequences against NCBI nr and UniProt DBs, respectively, and retrieved up to 20 best hits based on alignment score. For taxonomic annotation, mPies returned the last common ancestor (LCA) among the best NCBI hits via MEGAN (bit score >80) [43] (File S3). For functional annotation, mPies returned the most frequent protein name, with a consensus tolerance threshold above 80% similarity amongst the 20 best UniProt hits. Proteins annotated with a score below this threshold were manually validated (File S4).

## 3. Results

### 3.1. Diel Structure of the Microbial Communities

**Figure 1:**
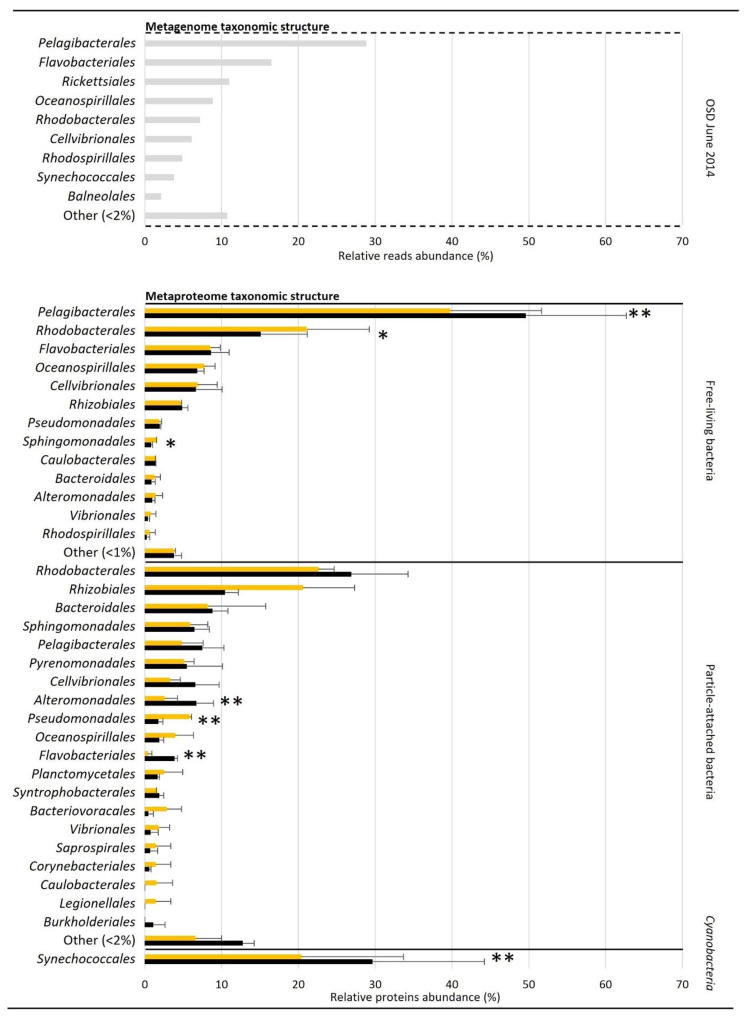
Order distribution of the bacterial communities obtained by metagenomics, and diel variability of active orders obtained by metaproteomics. Metagenomic data consisted of the percentage of total 16S rRNA reads observed over the OSD14 sampling effort (day for 0.2 µm pore-sized fraction). Metaproteomic data consisted of the average percentage of total unique peptide spectra detected per order for each metaproteome (day (yellow) and night (black) for both 0.2 and 0.8 µm size-fractions, *n* = 2). The least abundant taxa (<2% of reads and <1 or 2% of peptide spectra) were classified in “Other” category. Significative differences between day and night samples are shown with a * (*p* value ≤ 0.1) or ** (*p* value ≤ 0.05) and were calculated with a paired *t*-test.

The reads encoding for the 16S rRNA were extracted from the OSD14 metagenome and reflected the abundance of each operational taxonomic unit (OTU) in the studied bacterial communities. The metagenome taxonomic structure showed that *Proteobacteria* was the most abundant phylum, with 66.89% of the total detected 16S rRNA bacterial reads, followed by *Bacteroidetes* (15.51%) and *Cyanobacteria* (12.22%) (Table 1). *Alphaproteobacteria* was the class with the highest representation (47.35%), followed by *Gammaproteobacteria* (17.77%), *Flavobacteriia* (14.32%) and unclassified *Cyanobacteria* (12.33%) (Table 1). At order level, *Pelagibacterales* reads were dominant (28.85%), followed by *Flavobacteriales* (16.48%) and, to a lesser extent, *Rickettsiales* (10.99%), *Oceanospirillales* (8.85%), *Rhodobacterales* (7.17%) and *Cellvibrionales* (6.10%) (Figure 1).

The metaproteomic analyses were performed on duplicate day and night samples and showed that the average number of identified bacterial proteins on the four 0.2 µm pore-sized filters was 550 ± 49 in the day (*n* = 2) and 452 ± 4 at night (*n* = 2) (Table 1). The active taxa of this bacterial fraction were largely characterized as *Proteobacteria* (average relative protein abundance during the day (D): 89.34 ± 1.62%, during the night (N): 92.43 ± 1.19%), followed by *Bacteroidetes* (D: 6.48 ± 0.61%, N: 5.48 ± 0.40%). Few *Cyanobacteria* proteins (D: 2.30 ± 1.83%, N: 0.43 ± 0.15%) were also observed (Table 1). At class level, *Alphaproteobacteria*, *Gammaproteobacteria* and *Flavobacteriia* were found to be the most represented (Table 1). At order level, *Pelagibacterales* proteins (D: 39.8 ± 11.9%, N: 49.6 ± 13.1%) were dominant, especially during the night (*p* value = 0.03), followed by *Rhodobacterales* (D: 21.1 ± 8.1%, N: 15.1 ± 6.1%). *Rhodobacterales* and *Sphingomonadales* proteins were represented more during the day (*p* values = 0.08 and 0.07, respectively) (Figure 1).

On the four 0.8 µm pore-sized filters, an average of 123 ± 28 (*n* = 2) and 170 ± 28 (*n* = 2) bacterial proteins were identified at day and at night, respectively (Table 1). *Cyanobacteria* (D: 60.25 ± 7.55%, N: 60.83 ± 6.60%) were the most abundant active players of this fraction, followed by *Proteobacteria* (D: 34.75 ± 7.92%, N: 33.19 ± 6.02%). Classes were mainly represented by unclassified *Cyanobacteria* and, to a lesser extent, by *Alphaproteobacteria* and *Gammaproteobacteria* (Table 1). At order level, *Rhodobacterales* proteins (D: 22.6 ± 2.1%, N: 26.9 ± 7.4%) were dominant during the day and the night (Figure 1). Proteins from *Pseudomonadales* were more abundant in the day (*p* value = 0.05), and *Alteromonadales* and *Flavobacteriales* at night (*p* values = 0.03 and 0.01, respectively) (Figure 1).

*Cyanobacteria* were more represented on the 0.8 µm pore-sized filters (D: 60.52 ± 8.06%, N: 60.83 ± 6.60%) compared to the 0.2 µm pore-sized filters (D: 2.30 ± 1.83%, N: 0.43 ± 0.15%) (Table 1), due to their rod-shaped morphology (>0.2 µm in length) [44]. Therefore, all identified cyanobacterial proteins were grouped for clarity purposes and compared with free-living and particle-attached bacteria (Figure 1). *Cyanobacteria* were exclusively characterized as *Synechococcales*, which represented 20.4 ± 13.3% (D) and 29.6 ± 14.6% (N) of the total identified proteins in both 0.2 and 0.8 µm fractions (Figure 1). The total *Synechococcales* proteins were significantly more abundant at night (*p* value = 0.03).

### 3.2. Diel Functioning of the Microbial Communities

The proteins characterized in the eight metaproteomes were grouped into five functional categories: (i) Protein folding and stress response, (ii) energy metabolism and compound biosynthesis, (iii) replication, transcription, and translation, (iv) transport and (v) cell mobility, structure, and division. Overall, the protein functions detected in the free-living bacterial community were found to be stable despite the diel variation, with only two proteins—the glyceraldehyde-3-phosphate dehydrogenase and the actin-like protein—being significantly more represented at day and at night, respectively (Table 2). In contrast, the proteins expressed by the particle-attached bacteria and *Cyanobacteria* showed more important diel changes, mainly in energy metabolism and compound biosynthesis processes (Table 2).

#### 3.2.1. Protein Folding and Response to Stress

**Figure 2:**
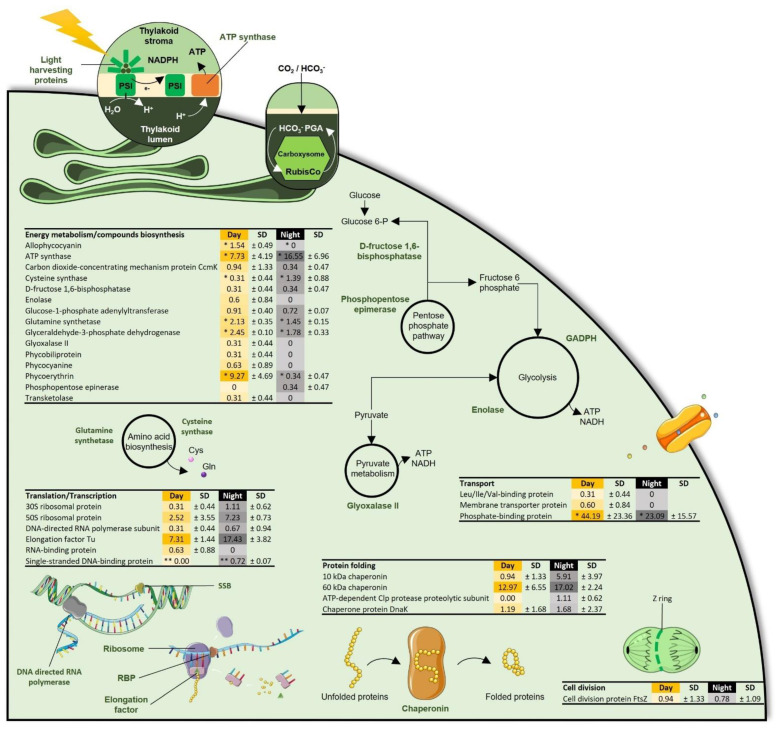
Representation of the cellular processes in *Synechococcales* revealed by metaproteomic analyses. Values consisted of the average percentage of total unique peptide spectra detected per protein function detected during day (yellow, *n* = 2) and night (black, *n* = 2) in all *Synechococcales* characterized in the 0.2 and 0.8 µm fractions. Significative differences between day and night samples are shown with a * (*p* value ≤ 0.1) or ** (*p* value ≤ 0.05) and were calculated with a paired *t*-test.

**Figure 3:**
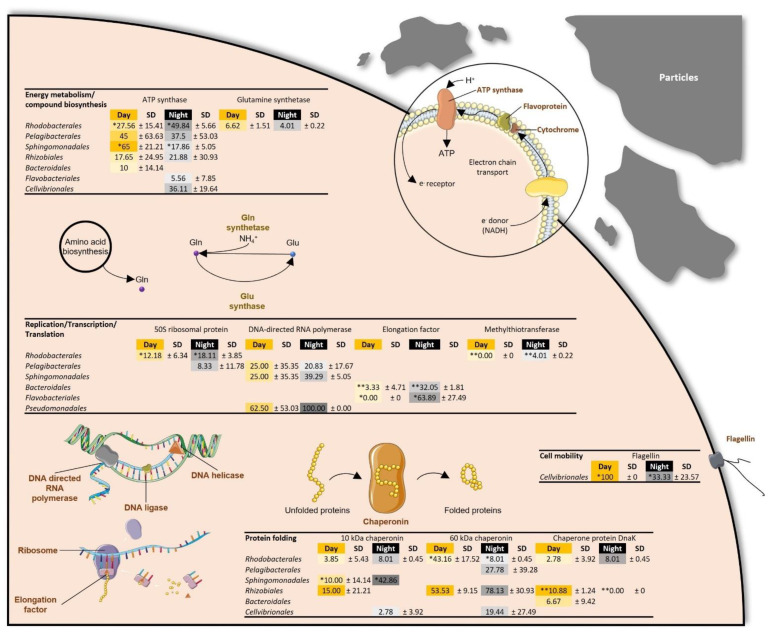
Representation of the cellular processes in particle-attached bacteria revealed by metaproteomic analyses. Values consisted of the average percentage of total unique peptide spectra detected per protein function detected during day (yellow, *n* = 2) and night (black, *n* = 2) in all particle-attached bacteria characterized in the 0.8 µm fractions. Significant differences between day and night samples are shown with a * (*p* value ≤ 0.1) or ** (*p* value ≤ 0.05) and were calculated with a paired *t*-test.

**Figure 4:**
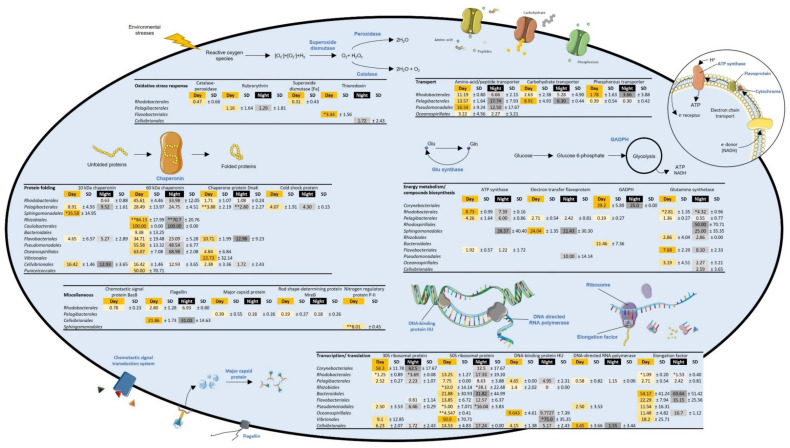
Representation of the cellular processes in free-living bacteria revealed by metaproteomic analyses. Values consisted of the average percentage of total unique peptide spectra detected per protein function detected during day (yellow, *n* = 2) and night (black, *n* = 2) in all particle-attached bacteria characterized in the 0.2 µm fractions. Significant differences between day and night samples are shown with a * (*p* value ≤ 0.1) or ** (*p* value ≤ 0.05) and were calculated with a paired t-test.

Proteins involved in protein folding were detected in the eight metaproteomes, with the 60 kDa chaperonin being the major protein function, followed by the 10 kDa chaperonin and the chaperone protein DnaK (Table 2). While the protein folding process was equally expressed in the *Cyanobacteria Synechococcales* (Figure 2), diel variations in chaperonin expression were observed in some free-living and particle-attached bacterial orders: during the day, the 60 kDA chaperonin was more abundant in particle-attached *Rhodobacterales* (*p* value = 0.10) and in free-living *Rhizobiales* (*p* value = 0.04), and the chaperone protein DnaK was more represented in particle-attached *Rhizobiales* (*p* value = 0.02) and free-living *Pelagibacterales* (*p* value = 0.02) (Figure 3 and Figure 4). Interestingly, the 10 kDa chaperonin was found to be differentially regulated over time among free-living and particle-attached *Sphingomonadales* (*p* values = 0.09 and 0.09, respectively), as the trends in protein abundance were higher during the day and night, respectively (Figure 3 and Figure 4).

Proteins involved in stress response processes were exclusively characterized in a few free-living bacterial orders, including *Rhodobacterales*, *Pelagibacterales*, *Flavobacteriales* and *Cellvibrionales* (Table 2, Figure 4). Interestingly, the catalase-peroxidase and superoxide dismutase [Fe], which both take part to the oxidative stress response, were exclusively detected during the day in *Rhodobacterales* (Figure 4). In contrast, the thioredoxin was only observed during the day in *Flavobacteriales* and during the night in *Cellvibrionales* (Figure 4), while the rubrerythrin and the cold-shock protein were expressed by *Pelagibacterales* during both the day and the night (Figure 4).

#### 3.2.2. Replication, Transcription, and Translation

Replication, transcription, and translation processes, mainly represented by the DNA-directed RNA polymerase, the 30S and 50S ribosomal proteins and the elongation factor, were characterized in both day and night metaproteomes (Table 2). These biological processes were equally represented during the day and at night in *Synechococcales* (Figure 2). Within the attached bacterial fraction, translational proteins (i.e., 50S ribosomal protein and elongation factor) were over-represented at night in *Rhodobacterales*, *Bacteroidales* or *Flavobacteriales*, while the abundance of the DNA-directed RNA polymerase was similar during both day and night (Figure 3). The 30S and 50S ribosomal proteins, the elongation factor, the DNA-binding protein HU and the DNA-directed RNA polymerase were also characterized in numerous free-living bacterial orders (Figure 4). These proteins were not impacted by the diel cycle except in *Rhodobacterales*, *Rhizobiales* and *Pseudomonadales*, where the translation process seemed more important during the night (Figure 4).

#### 3.2.3. Energy Metabolism and Compounds Biosynthesis

Energy metabolism and compound biosynthesis accounted for the most diverse functional category and was particularly represented in the particle-attached bacteria and in *Cyanobacteria* metaproteomes (Table 2). The ATP synthase was the dominant protein and showed diel variation in both the particle-attached bacterial community (*p* value = 0.02) and in *Cyanobacteria* (*p* value = 0.07), while it was stable in the free-living bacterial fraction (Table 2). Other proteins related to the energy metabolism and involved in the pentose phosphate pathway, the glycolysis or the pyruvate metabolism were found to be regulated over time in *Cyanobacteria* (Table 2). Photosynthesis proteins such as the phycoerythrin and the allophycocyanin were clearly synchronized with daytime in *Synechococcales* (*p* value = 0.10 and 0.07, respectively) (Figure 2).

#### 3.2.4. Transport and Cell Division, Structure, and Mobility

Interestingly, the amino acid biosynthesis pathway was represented in all metaproteomes by the glutamine synthase, involved in glutamine metabolism via the incorporation of ammonium ion into glutamate [45] (Table 2). This protein displayed diel variations in *Synechococcales* (*p* value = 0.07) and in free-living *Rhodobacterales* (*p* value = 0.06), where it was dominant at day and night, respectively (Figure 2 and Figure 4). In *Synechococcales*, the amino acid biosynthesis pathway was also characterized by the cysteine synthase, which showed contrasting diel variability with the glutamine synthase as it peaked at night (*p* value = 0.09) (Figure 2).

Numerous amino acid/peptides, carbohydrate and phosphorous transporters were observed in free-living bacterial metaproteomes (Figure 4). The phosphorous transporters were the major proteins identified in *Cyanobacteria* (Figure 2). In contrast, no transport related protein was detected in the particle-attached fraction. While the phosphorous transporters in *Synechococcales* were over expressed during the day (*p* value = 0.08) (Figure 2), no diel variation was observed in transporter abundance of free-living bacteria (Figure 4).

Among the last protein functions observed, the cell division protein (FtsZ) was present in *Synechococcales* metaproteomes, and its expression was not affected by the diel cycle (Figure 2). Flagellin protein, expressed by *Cellvibrionales*, showed contrasting diel regulation depending on the bacterial lifestyle as it was stable over time in the free-living fraction and peaked during the day in the particle-attached fraction (*p* value = 0.08) (Figure 3 and Figure 4). This protein was also detected in free-living *Rhodobacterales* where no diel variation was observed (Figure 4). However, proteins associated with the chemotaxis system in free-living *Rhodobacterales* were found to express at day only (*p* value = 0.07) (Figure 4). Finally, the major capsid protein and the rod shape-determining protein MreB were characterized in free-living *Pelagibacterales* during both the day and the night (Figure 4).

## 4. Discussion

This study compared day and night protein abundance between free-living and particle-attached bacteria from an oligotrophic marine surface environment. Metaproteomic analyses were performed on duplicate filters for both conditions (day/night) and pore-sized fractions (0.2/0.8 µm). Although we appreciate that more replicates could be performed, the low standard deviation of our samples allowed us to provide the first overview of protein diel variations at the sea surface. The total identified proteins of both bacterial fractions were consistent with previous metaproteomic studies conducted in marine oligotrophic surface waters [25,46,47,48] (Table 1). The taxonomic distribution of the OSD14 metagenome showed that the most abundant members of the community were *Proteobacteria*, followed by *Bacteroidetes* and *Cyanobacteria* (Table 1). These taxa were previously reported as numerically abundant in coastal marine oligotrophic environments, such as the Mediterranean Sea [49], the Antarctic [47,48] and Atlantic [29,50] surface waters. The metaproteome taxonomic structure was found to be similar to that of OSD14 metagenome (Table 1), indicating a correlation between abundant vs. metabolically active community members. Moreover, free-living and particle-attached metaproteomes showed high similarities in taxonomic distribution. Overlaps in the structure of both bacterial fractions were previously reported within microbial assemblages of the Mediterranean Sea, where the colonization of particles was suggested to be largely mediated by free-living bacteria present in the surrounding water [9,10,11].

Interestingly, the representation of *Cyanobacteria* was higher in the metaproteomes than in the OSD14 metagenome (Table 1). All identified *Cyanobacteria* proteins were classified as *Synechococcales* (Figure 1), which is a main contributor to the primary production in oligotrophic water during summer [51]. In contrast, *Bacteroidetes*, mainly characterized as *Flavobacteriia*, were less represented in the metaproteomes (Table 1). *Flavobacteriia* are bloom-associated bacteria known for degrading phytoplankton-derived compounds [52]. The NW Mediterranean Sea is characterized by spring and autumnal phytoplankton blooms separated by an oligotrophic summer [53], which could explain why this group was less abundant at the protein level at the sampling time. 

Comparison of day and night metaproteomes revealed differences between free-living and particle-attached bacteria (Figure 1). Proteins of free-living bacteria were the most represented in all samples and peaked during the day, while proteins expressed by particle-attached bacteria showed higher abundance during the night (Table 1). Similar observations were reported for the bacterial activity measured by ^3^H-leucine incorporation in the NW Mediterranean Sea in summer [11]. The activity of attached bacteria depends on the nature and concentration of aggregates and suspended particles, which represent hot-spots for microbial processes [5]. In the upper layer of the NW Mediterranean Sea during summer, the release of organic material from photosynthetic microorganisms and zooplankton was suggested to be a major factor driving the diel variation in particle-attached bacterial activity [11]. In this study, metaproteomic analyses showed that particle-attached *Flavobacteriales* proteins were more abundant at night (Figure 1). Therefore, the representation of *Flavobacteriales* could increase at night in response of zooplankton feeding on phytoplankton and releasing organic matter [11].

Time-keeping mechanisms in *Synechococcales* are well described and show that circadian clock regulates patterns of genetic expression throughout the day using external variable clues (e.g., light, temperature and/or redox cycles) to scale to the environment [54]. Distinct diel profiles of protein abundance were observed between metaproteomes in *Synechococcales* (Figure 1). Proteins involved in photosynthesis and phosphate transport showed a clear trend in abundance that was higher in the day than at night (Table 2, Figure 2). These results confirmed previous comparative day/night (meta)-transcriptomic studies that showed a higher abundance of transcripts for photosynthesis during the day compared with the night [18,20]. In contrast, proteins involved in housekeeping functions such as protein folding, translation, transcription, and cell division displayed similar abundances in both day and night samples (Table 2, Figure 2). Proteins involved in catabolic pathways including glycolysis, pyruvate metabolism and respiration were also observed in similar abundance in the day and at night, except for the ATP synthase, which was twice more represented during the night (Figure 2). This suggested that *Synechococcales* maintained housekeeping activity independently of diel variation but increased ATP production during the night when photosynthesis is shut down. 

Chaperonin proteins, which are characterized as ubiquitous in many marine ecosystems [25,48,55], were highly represented in both day and night community metaproteomes (Table 2). During summer, bacteria in the euphotic layer are exposed to high UV radiation, altering both proteins and DNA structure. Mechanisms such as protein folding, reactive oxygen species reduction and protein biosynthesis are essential for coping with protein damage and maintaining proper cellular functions [56]. Chaperonin abundance was not impacted by diel cycle in most bacterial orders, with a few exceptions including *Rhodobacterales*, *Sphingomonadales*, *Rhizobiales* and *Pelagibacterales* (Figure 2 and Figure 3). Proteins involved in protein biosynthesis (i.e., ribosomal protein and elongation factor) were more abundant at night in free-living and particle-attached *Rhodobacterales* (Figure 3 and Figure 4). Interestingly, proteins involved in oxidative stress response such as the catalase-peroxidase and the superoxide dismutase [Fe] were only observed during the day in free-living *Rhodobacterales* (Figure 4). Thioredoxin was exclusively detected in the day in *Flavobacteriales* and at night in *Cellvibrionales* (Figure 4). In contrast, rubrerythrin and cold shock protein were present in *Pelagibacterales* during both the day and night (Figure 4). These observations suggested that protein regulation in response to environmental stress is taxa-specific and depends on lifestyle (free-living vs. particle-attached). Protein regulation in a protein repair system could be time-gated in bacterial orders such as *Rhodobacterales* or constitutive in other such as *Pelagibacterales*. 

Proteins involved in compounds transport were detected in free-living bacteria and *Cyanobacteria* during both the day and the night (Table 2). Glutamine synthetase, involved in nitrogen metabolism, was detected in all bacterial fractions (Table 2, Figure 2 and Figure 4). The abundance of transporters in the free-living fraction and the overall characterization of glutamine synthetase suggested an adaptation to an oligotrophic environment, where a strong competition for limiting nutrients such as nitrogen and phosphorous was reported [14,26,57]. Interestingly, no transporter was identified in the attached-bacterial fraction (Table 2, Figure 3). This suggested lesser environmental pressure for the expression of nutrient transporters in attached bacteria since nutrients are more readily available in the particle microenvironment. In contrast, free-living bacteria and *Cyanobacteria* could depend on constitutive expression of transporters for efficient nutrient scavenging [26].

*Pelagibacterales* dominated the free-living bacterial community, in both the metagenome and the metaproteomes (Figure 1). *Pelagibacterales* include proteorhodopsin-containing photoheterotrophs such as *Pelagibacter* (SAR11), which is known to be abundant and highly active in the ocean [46]. Metatranscriptomic studies showed evidence of diel periodicity in many of their gene transcripts [20]. Here, proteins involved in protein folding, stress response and replication, transcription and translation were the main functions characterized in *Pelagibacterales* metaproteomes (Figure 3 and Figure 4). Despite the higher trend in protein abundance of free-living *Pelagibacterales* at night (Figure 1), no significant change was observed in the aforementioned biological processes (Figure 1). This suggests that *Pelagibacterales* constitutively express diverse housekeeping genes required for the maintenance of basal cellular functions that are essential to protect the cell against molecular damage and environmental changes. Protein expression regulation could take place at transcript level, thus limiting energy losses from diel protein turnover [58].

## 5. Conclusions

This study provided the first overview on the picoplanktonic response to diel variation at the protein level and demonstrated taxa-specific diel protein regulation from surface marine microbial communities. Taxonomic overlaps were observed between free-living and particle-attached bacteria, where protein abundance peaked at day and at night, respectively. The photoautotrophs *Synechococcales* showed distinct diel protein profiles with light-dependent functions synchronized with daytime. Similarly, diel variations in (photo)-heterotrophic bacteria were observed, thus revealing distinct adaptation strategies with essential regulations in environmental stress response. This study provided preliminary results reinforcing the hypothesis that the functioning of free-living and particle-attached communities could be time-gated. Additional work, including observational studies with more sampling replicates and laboratory-based investigations, is needed to further understand the response of these communities to diel changes and to decipher the cellular mechanisms involved in the diel adaptation of (photo)-heterotroph microorganisms.

## Figures and Tables

**Table 1:** Structure of the bacterial communities obtained by metagenomic and major bacterial players—active taxa—obtained by metaproteomics at phylum and class levels. Metagenomic data consisted of the percentage of total 16S rRNA bacterial reads observed over the OSD14 sampling effort (day for 0.2 µm pore-sized fraction). Metaproteomic data consisted of the average percentage of total unique bacterial peptide spectra detected per phylum or class for each metaproteome (day (yellow) and night (black) for both 0.2 and 0.8 µm pore-sized fractions, *n* = 2). The least abundant taxa (<1% of total reads or peptide spectra) were classified in “Other” category. Significative differences between day and night samples are shown with a * (*p* value ≤ 0.1) or ** (*p* value ≤ 0.05) and were calculated with a paired *t*-test.

	Metagenome Taxonomic Structure	Metaproteome Taxonomic Structure							
		0.2 µm Size-Fraction				0.8 µm Size-Fraction			
	OSD June 2014	Day	SD	Night	SD	Day	SD	Night	SD
Total reads/proteins	761	550	±49	452	±4	123	±28	170	±28
**Phylum**									
*Proteobacteria*	66.89	** 89.34	±1.62	** 92.43	±1.19	34.75	±7.92	33.19	±6.02
*Bacteroidetes*	15.51	6.48	±0.61	5.48	±0.40	2.71	±1.35	4.47	±0.84
*Cyanobacteria*	12.22	2.30	±1.83	0.43	±0.15	60.52	±8.06	60.83	±6.60
*Rhodothermaeota*	1.84	0.69	±0.22	0.56	±0.61				
*Planctomycetes*	0.13			0.03	±0.05	0.75	±0.79	0.46	±0.03
Other (<1%)	3.42	1.19		1		1.26		1.05	
**Class**									
*Alphaproteobacteria*	47.35	* 67.06	±2.88	* 71.54	±4.87	20.88	±2.04	20.86	±4.04
*Gammaproteobacteria*	17.77	23.29	±0.69	21.57	±4.01	12.79	±4.64	11.41	±0.97
*Flavobacteriia*	14.32	4.77	±0.75	4.94	±0.75	** 0.10	±0.14	** 1.46	±0.10
Unclassified *Cyanobacteria*	12.33	0.54	±0.77	0.45	±0.16	60.25	±7.55	60.26	±5.63
*Bacteroidia*	0.13	0.68	±0.47	0.51	±0.32	2.27	±1.95	2.65	±0.86
*Deltaproteobacteria*		0.06	±0.08			* 0.43	±0.03	* 0.78	±0.21
*Oligoflexia*		0.06	±0.09	0.07	±0.09	0.87	±0.66	0.44	±0.29
*Planctomycetia*				0.03	±0.05	0.77	±0.80	0.49	±0.03
Other (<1%)	8.09	3.54		0.9		1.64		1.64	

**Table 2:** Diel variation in protein function abundances in free-living and particle-attached bacterial fraction and *Cyanobacteria* revealed by metaproteomics. Values consisted of the average percentage of total unique peptide spectra detected per protein function in free-living bacteria, particle-attached bacteria, and all *Cyanobacteria* during both day (yellow, *n* = 2) and night (black, *n* = 2). The least abundant functions (<1% of peptide spectra) were classified in “Other” category. Significative differences between day and night samples are shown with a * (*p* value ≤ 0.1) or ** (*p* value ≤ 0.05) and were calculated with a paired *t*-test.

	Free-Living Bacteria				Particle-Attached Bacteria				*Cyanobacteria*			
**Protein folding/ Stress response**	**Day**	**SD**	**Night**	**SD**	**Day**	**SD**	**Night**	**SD**	**Day**	**SD**	**Night**	**SD**
10 kDa chaperonin	5.36	±0.22	5.52	±0.74	3.48	±1.66	4.64	±1.32	0.94	±1.33	5.91	±3.97
60 kDa chaperonin	32.46	±1.19	30.29	±5.42	** 20.54	±6.29	** 25.52	±6.96	13.02	±6.48	17.02	±2.24
ATP-dependent Clp protease proteolytic subunit											1.11	±0.62
Chaperone protein DnaK	3.29	±0.04	3.72	±0.62	5.77	±3.22	4.01	±1.01	1.20	±1.70	1.68	±2.37
Cold shock protein	0.66	±0.05	0.97	±0.31								
Rubrerythrin	1.35	±0.17	1.98	±0.84								
**Energy metabolism/Compounds biosynthesis**												
ATP synthase	3.43	±0.23	2.97	±0.01	** 18.23	±6.28	** 11.68	±5.59	* 7.75	±4.15	* 16.55	±6.96
Aconitate hydratase B	0.12	±0.16			1.74	±2.46						
Cysteine synthase									* 0.31	±0.44	* 1.39	±0.87
Fructose-1,6-bisphosphatase							1.72	±2.42				
Glutamine synthetase	2.56	±0.21	2.17	±0.44	** 1.44	±1.21	** 1.27	±1.22	*2.14	±0.36	* 1.44	±0.14
Glyceraldehyde-3-phosphate dehydrogenase	* 0.20	±0.04	* 0.09	±0.00	3.77	±3.70	4.35	±4.32	*2.46	±0.07	* 1.78	±0.32
Isocitrate dehydrogenase [NADP]					0.87	±1.23	2.49	±2.30				
Molybdopterin molybdenumtransferase					1.73	±0.80	0.41	±0.58				
Phycoerythrin									* 9.30	±4.63	* 0.33	±0.47
Allophycocyanin									* 1.54	±0.48	* 0	
Carbon dioxide-concentrating mechanism protein CcmK	0.04	±0.05	0.05	±0.07					0.94	±1.33	0.34	±0.47
Formate dehydrogenase	0.72	±0.40	0.74	±0.91								
Glucose-1-phosphate adenylyltransferase									0.31	±0.44	0.72	±0.07
**Replication/Transcription/Translation**												
30S ribosomal protein	2.91	±0.39	2.66	±0.81					0.31	±0.44	1.11	±0.62
50S ribosomal protein	13.47	±1.37	12.86	±4.44	2.32	±1.65	10.40	±7.14	2.52	±3.55	7.23	±0.73
DNA-binding protein HU	7.16	±0.09	7.69	±0.63								
DNA-directed RNA polymerase	0.54	±0.10	0.76	±0.23	7.77	±8.51	9.52	±4.73	0.31	±0.44	0.67	±0.94
Elongation factor	5.62	±0.47	6.77	±2.98	6.94	±0.05	9.40	±3.58	7.36	±1.51	17.43	±3.82
Histone-like protein	0.16	±0.10	0.15	±0.07	* 11.25	±5.22	* 3.80	±1.89				
Glycine-tRNA ligase					1.44	±1.21	1.25	±0.55				
Ribosomal protein S12 methylthiotransferase RimO							1.07	±1.51				
**Transport**												
Amino-acid ABC transporter-binding protein	5.61	±0.74	6.31	±0.36								
Fructose import binding protein FrcB	1.13	±0.50	1.26	±0.60								
Phosphate-binding protein	0.28	±0.15	0.29	±0.28					44.56	±23.88	23.09	±15.58
**Cell motility, structure, and division**												
Cell division protein FtsZ									0.94	±1.33	0.78	±1.09
Actin-like protein	* 0.70	±0.23	* 0.90	±0.15								
Tubulin					3.77	±2.07	2.57	±3.04				
Peptidoglycan-associated lipoprotein	0.37	±0.02	0.75	±0.63								
Flagellin	4.26	±0.41	5.37	±0.40	4.91	±1.18	3.58	±0.40				
Other (<1%)	7.60		5.72		4.03		2.30		4.03		1.40	

## Data Availability

The metaproteomic raw data are available in the iProx public platform (Project ID: IPX0002008000; Subproject IDs: IPX0002008001 (free-living fractions), IPX0002008002 (particle-attached fractions)). The physicochemical data are available from SOMLIT on request. The metagenomic data are available from EBI (Project number: ERP009703, Ocean Sampling Day 2014, sample: OSD14_2014_06_2m_NPL022, run ID: ERR771073).

## Supplementary Materials

The following are available online at www.mdpi.com/article/10.3390/microorganisms9122621/s1, File S1: protein identification summary statistics reports (ProteinPilot^TM^); File S2: FDR analysis reports (ProteinPilot™); File S3: taxonomic protein annotation reports (mPies v. 0.9); File S4: functional protein annotation reports (mPies v. 0.9).
