# Supplementary material for: Diel Protein Regulation of Marine Picoplanktonic Communities Assessed by Metaproteomics
Source: Microorganisms. 2021 Dec 18;9(12):2621. doi: 10.3390/microorganisms9122621 (PMC8707726; doi:10.3390/microorganisms9122621)
Supplement: Supplementary file 1 [file microorganisms-09-02621-s001.zip › File S1. Summary_statistics_report_ProteinPilotTM.pdf]

Sample name: 8jSET18 - Metaproteome name: particle-attached day 1

ProteinPilot™ Software - [Result - S:\Augustin\ProteinPilot\2rdDB_08_rerun\8jSET18(1).group]

File Configure Window Help

Workflow Tasks

Identify Proteins
LC...
Spot-Based (MS only)...
Spot-Based (MS and MS/MS)...

View
Analysis Log...
Result...

Export
Peptide Summary...
Distinct Peptide Summary...
Protein Summary...
Spectrum Summary...
MGF Peaklist(s)...
mzIdentML...
Features...

Export the features to a text file

Protein ID | Features | Spectra | Summary Statistics

ID Statistics (Protein-Thresholded): 88177 total spectra, 88177 non-empty spectra; 20038 proteins searched

Unused (Conf) Cutoff	Proteins Detected	Proteins Before Grouping	Distinct Peptides	Spectra Identified	% Total Spectra
>2.0 (99)	43	3271	331	1083	1.2
>1.3 (95)	85	5031	386	1387	1.6
>0.47 (66)	99	5519	413	1440	1.6
Cutoff Applied: >0.05 (10%)	161	6005	530	1621	1.8

Result Parameters

Detected Protein Threshold [Unused ProtScore (Conf)] >: 0.05 (10.0%)
Competitor Error Margin (ProtScore): 2.00
Software Version: ProteinPilot™ Software 5.0.1
Revision Number: 4895
Paragon™ Algorithm: 5.0.1.0, 4874
Annotations Retrieved from UniProt: No

Analysis Parameters

1) S:\Augustin\Wiff\8jSET18.wiff

Sample Type: Identification
Cys. Alkylation: Iodoacetamide
Digestion: Trypsin
Instrument: TripleTOF 5600
Special Factors:
Species:
ID Focus: Biological modifications
Amino acid substitutions
Database: 08_Second_round_DB.fasta
Search Effort: Thorough
FDR Analysis: Yes
User Modified Parameter Files: Yes

Sample name: 8nSET19 - Metaproteome name: particle-attached night 1

ProteinPilot™ Software - [Result - S:\Augustin\ProteinPilot\2rdDB_08_rerun\8nSET19.group]

File Configure Window Help

Workflow Tasks

Identify Proteins
LC...
Spot-Based (MS only)...
Spot-Based (MS and MS/MS)...

View
Analysis Log...
Result...

Export
Peptide Summary...
Distinct Peptide Summary...
Protein Summary...
Spectrum Summary...
MGF Peaklist(s)...
mzIdentML...
Features...

Protein ID | Features | Spectra | Summary Statistics

ID Statistics (Protein-Thresholded): 87643 total spectra, 87643 non-empty spectra; 20038 proteins searched

Unused (Conf) Cutoff	Proteins Detected	Proteins Before Grouping	Distinct Peptides	Spectra Identified	% Total Spectra
>2.0 (99)	79	3769	572	2184	2.5
>1.3 (95)	141	7495	657	2585	2.9
>0.47 (66)	155	7706	689	2656	3.0
Cutoff Applied: >0.05 (10%)	182	7775	729	2742	3.1

Result Parameters

Detected Protein Threshold [Unused ProtScore (Conf)] >: 0.05 (10.0%)
Competitor Error Margin (ProtScore): 2.00
Software Version: ProteinPilot™ Software 5.0.1
Revision Number: 4895
Paragon™ Algorithm: 5.0.1.0, 4874
Annotations Retrieved from UniProt: No

Analysis Parameters

1) S:\Augustin\Wiff\8nSET19.wiff

Sample Type: Identification
Cys. Alkylation: Iodoacetamide
Digestion: Trypsin
Instrument: TripleTOF 5600
Special Factors:
Species:
ID Focus: Biological modifications
Amino acid substitutions
Database: 08_Second_round_DB.fasta
Search Effort: Thorough
FDR Analysis: Yes
User Modified Parameter Files: Yes

Sample name: 8j2SET110 - Metaproteome name: particle-attached day 2

ProteinPilot™ Software - [Result - S:\Augustin\ProteinPilot\2rdDB_08_rerun\8j2SET110.group]

File Configure Window Help

Workflow Tasks

Identify Proteins
- LC...
- Spot-Based (MS only)...
- Spot-Based (MS and MS/MS)...

View
- Analysis Log...
- Result...

Export
- Peptide Summary...
- Distinct Peptide Summary...
- Protein Summary...
- Spectrum Summary...
- MGF Peaklist(s)...
- mzIdentML...
- Features...

Protein ID | Features | Spectra | Summary Statistics

ID Statistics (Protein-Thresholded): 86844 total spectra, 86844 non-empty spectra; 20038 proteins searched

Unused (Conf) Cutoff	Proteins Detected	Proteins Before Grouping	Distinct Peptides	Spectra Identified	% Total Spectra
>2.0 (99)	81	2963	701	3033	3.5
>1.3 (95)	137	5495	786	3424	3.9
>0.47 (66)	150	5920	817	3492	4.0
Cutoff Applied: >0.05 (10%)	197	8745	900	3643	4.2

Result Parameters

Detected Protein Threshold [Unused ProtScore (Conf)] >: 0.05 (10.0%)
Competitor Error Margin (ProtScore): 2.00
Software Version: ProteinPilot™ Software 5.0.1
Revision Number: 4895
Paragon™ Algorithm: 5.0.1.0, 4874
Annotations Retrieved from UniProt: No

Analysis Parameters

1) S:\Augustin\Wiff\8j2SET110.wiff

Sample Type: Identification
Cys. Alkylation: Iodoacetamide
Digestion: Trypsin
Instrument: TripleTOF 5600
Special Factors:
Species:
ID Focus: Biological modifications
Amino acid substitutions
Database: 08_Second_round_DB.fasta
Search Effort: Thorough
FDR Analysis: Yes
User Modified Parameter Files: Yes

Sample name: 8n2SET111 - Metaproteome name: particle-attached night 2

ProteinPilot™ Software - [Result - S:\Augustin\ProteinPilot\2rdDB_08_rerun\8n2SET111.group]

File Configure Window Help

Workflow Tasks

Identify Proteins
- LC...
- Spot-Based (MS only)...
- Spot-Based (MS and MS/MS)...

View
- Analysis Log...
- Result...

Export
- Peptide Summary...
- Distinct Peptide Summary...
- Protein Summary...
- Spectrum Summary...
- MGF Peaklist(s)...
- mzIdentML...
- Features...

Protein ID | Features | Spectra | Summary Statistics

ID Statistics (Protein-Thresholded): 86537 total spectra, 86537 non-empty spectra; 20038 proteins searched

Unused (Conf) Cutoff	Proteins Detected	Proteins Before Grouping	Distinct Peptides	Spectra Identified	% Total Spectra
>2.0 (99)	108	4485	884	3791	4.4
>1.3 (95)	165	6638	977	4251	4.9
>0.47 (66)	188	6954	1018	4359	5.0
Cutoff Applied: >0.05 (10%)	235	7807	1104	4509	5.2

Result Parameters

Detected Protein Threshold [Unused ProtScore (Conf)] >: 0.05 (10.0%)
Competitor Error Margin (ProtScore): 2.00
Software Version: ProteinPilot™ Software 5.0.1
Revision Number: 4895
Paragon™ Algorithm: 5.0.1.0, 4874
Annotations Retrieved from UniProt: No

Analysis Parameters

1) S:\Augustin\Wiff\8n2SET111.wiff

Sample Type: Identification
Cys. Alkylation: Iodoacetamide
Digestion: Trypsin
Instrument: TripleTOF 5600
Special Factors:
Species:
ID Focus: Biological modifications
Amino acid substitutions
Database: 08_Second_round_DB.fasta
Search Effort: Thorough
FDR Analysis: Yes
User Modified Parameter Files: Yes

Sample name: 2jSET11 - Metaproteome name: free-living day 1

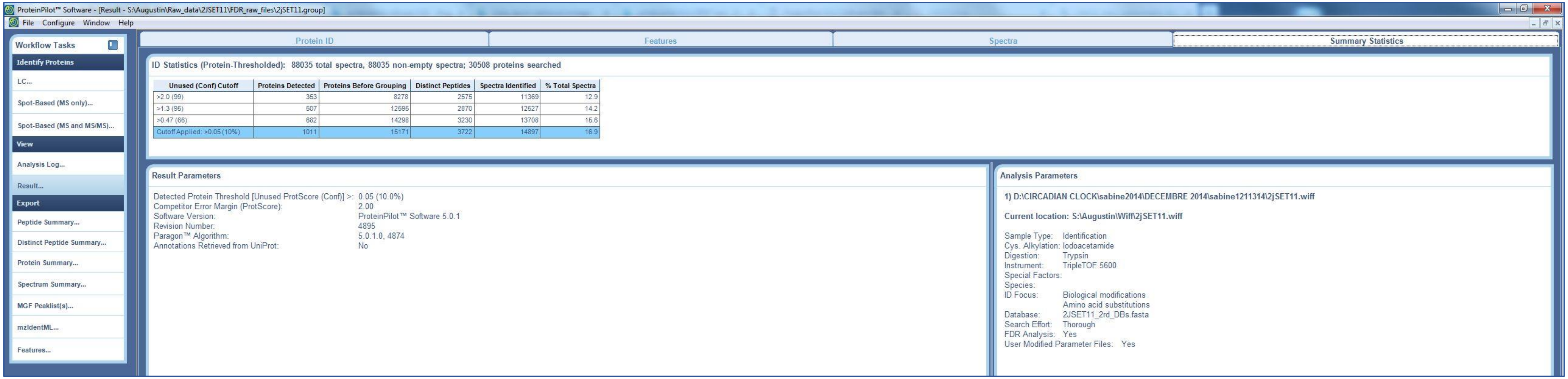

ID Statistics (Protein-Thresholded): 88035 total spectra, 88035 non-empty spectra; 30508 proteins searched

Unused (Conf) Cutoff	Proteins Detected	Proteins Before Grouping	Distinct Peptides	Spectra Identified	% Total Spectra
>2.0 (99)	353	8278	2575	11369	12.9
>1.3 (95)	507	12595	2870	12527	14.2
>0.47 (66)	682	14298	3230	13708	15.6
Cutoff Applied: >0.05 (10%)	1011	15171	3722	14897	16.9

Result Parameters

Detected Protein Threshold [Unused ProtScore (Conf)] >: 0.05 (10.0%)
Competitor Error Margin (ProtScore): 2.00
Software Version: ProteinPilot™ Software 5.0.1
Revision Number: 4895
Paragon™ Algorithm: 5.0.1.0, 4874
Annotations Retrieved from UniProt: No

Analysis Parameters

1) D:\CIRCADIAN CLOCK\sabine2014\DECEMBRE 2014\sabine1211314\2jSET11.wiff

Current location: S:\Augustin\Wiff\2jSET11.wiff

Sample Type: Identification
Cys. Alkylation: Iodoacetamide
Digestion: Trypsin
Instrument: TripleTOF 5600
Special Factors:
Species:
ID Focus: Biological modifications
Amino acid substitutions
Database: 2JSET11_2rd_DBs.fasta
Search Effort: Thorough
FDR Analysis: Yes
User Modified Parameter Files: Yes

Sample name: 2nSET12 - Metaproteome name: free-living night 1

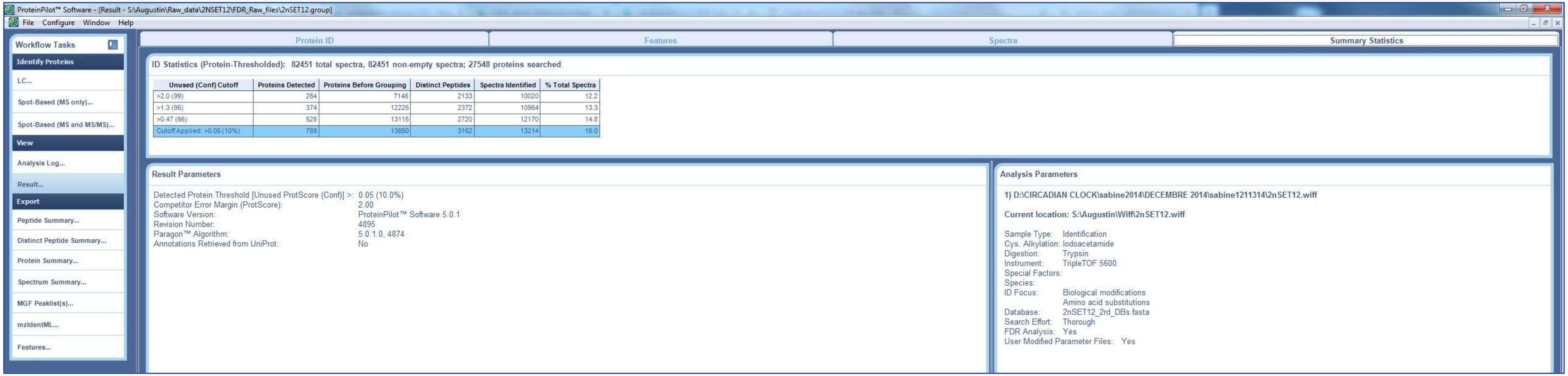

ID Statistics (Protein-Thresholded): 82451 total spectra, 82451 non-empty spectra; 27548 proteins searched

Unused (Conf) Cutoff	Proteins Detected	Proteins Before Grouping	Distinct Peptides	Spectra Identified	% Total Spectra
>2.0 (99)	264	7146	2133	10020	12.2
>1.3 (95)	374	12225	2372	10964	13.3
>0.47 (66)	528	13115	2720	12170	14.8
Cutoff Applied: >0.05 (10%)	788	13650	3162	13214	16.0

Result Parameters

Detected Protein Threshold [Unused ProtScore (Conf)] >: 0.05 (10.0%)
Competitor Error Margin (ProtScore): 2.00
Software Version: ProteinPilot™ Software 5.0.1
Revision Number: 4895
Paragon™ Algorithm: 5.0.1.0, 4874
Annotations Retrieved from UniProt: No

Analysis Parameters

1) D:\CIRCADIAN CLOCK\sabine2014\DECEMBRE 2014\sabine1211314\2nSET12.wiff

Current location: S:\Augustin\Wiff\2nSET12.wiff

Sample Type: Identification
Cys. Alkylation: Iodoacetamide
Digestion: Trypsin
Instrument: TripleTOF 5600
Special Factors:
Species:
ID Focus: Biological modifications
Amino acid substitutions
Database: 2nSET12_2rd_DBs.fasta
Search Effort: Thorough
FDR Analysis: Yes
User Modified Parameter Files: Yes

Sample name: 2j2SET13 - Metaproteome name: free-living day 2

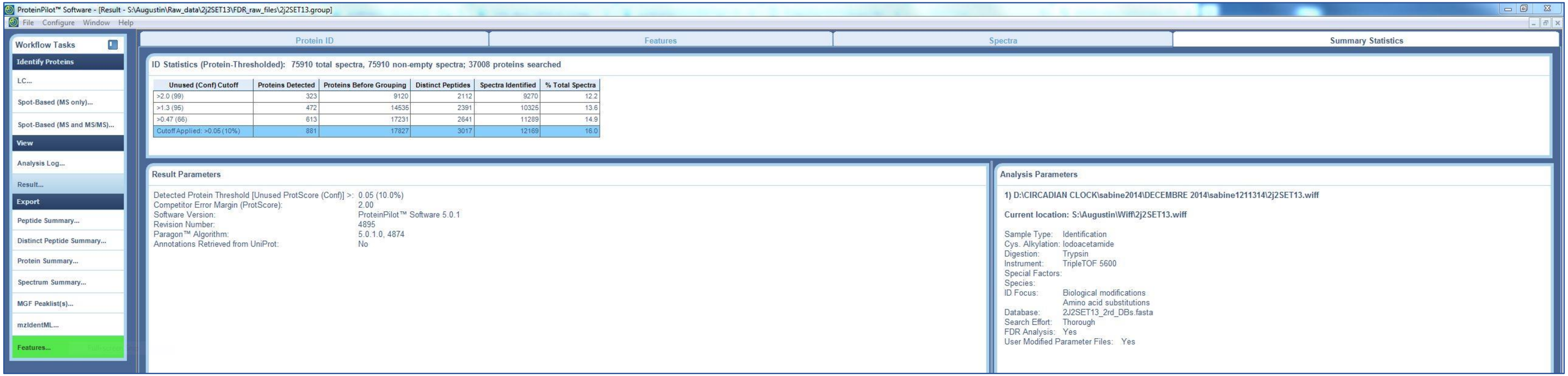

Unused (Conf) Cutoff	Proteins Detected	Proteins Before Grouping	Distinct Peptides	Spectra Identified	% Total Spectra
>2.0 (99)	323	9120	2112	9270	12.2
>1.3 (95)	472	14535	2391	10325	13.6
>0.47 (66)	613	17231	2641	11289	14.9
Cutoff Applied: >0.05 (10%)	881	17827	3017	12169	16.0

Sample name: 2n2SET14 - Metaproteome name: free-living night 2

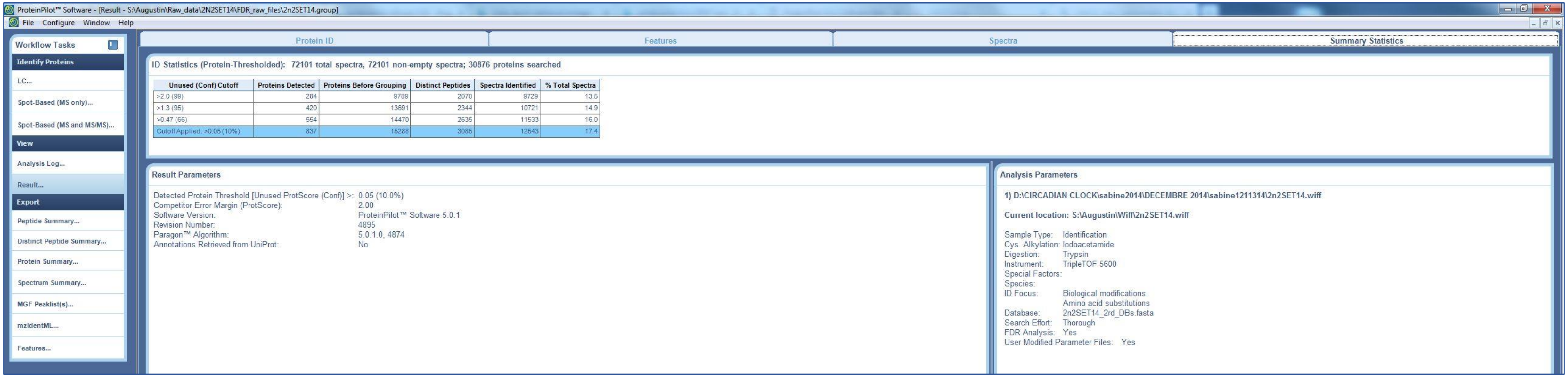

Unused (Conf) Cutoff	Proteins Detected	Proteins Before Grouping	Distinct Peptides	Spectra Identified	% Total Spectra
>2.0 (99)	284	9789	2070	9729	13.5
>1.3 (95)	420	13691	2344	10721	14.9
>0.47 (66)	554	14470	2635	11533	16.0
Cutoff Applied: >0.05 (10%)	837	15288	3085	12543	17.4
