# Supplementary material for: Diel Protein Regulation of Marine Picoplanktonic Communities Assessed by Metaproteomics
Source: Microorganisms. 2021 Dec 18;9(12):2621. doi: 10.3390/microorganisms9122621 (PMC8707726; doi:10.3390/microorganisms9122621)
Supplement: Supplementary file 1 [file microorganisms-09-02621-s001.zip › File S2. FDR_analysis_report_ProteinPilotTM.pdf]

Sample name: 2jSET11 - Metaproteome name: free-living day 1

ProteinPilot™ Software Report

Template Version 1.00 light report

Summary of Identification Yields

	Data Level	FDR Type	FDR	ID Yield
Identification Yield at FDR Threshold	Protein	Local	1%	514
			5%	569
			10%	603
		Global	1%	620
			5%	776
			10%	916
	Distinct peptide	Local	1%	888
			5%	1445
			10%	2017
		Global	1%	1368
			5%	2569
			10%	3847
	Spectral	Local	1%	4576
			5%	7115
			10%	9007
		Global	1%	6957
			5%	11435
			10%	14223
Corresponding Confidence in ProteinPilot™ Software	Protein	Local	1%	94.5%
			5%	88.3%
			10%	83.0%
		Global	1%	80.0%
			5%	42.5%
			10%	20.6%
	Distinct peptide	Local	1%	99.4%
			5%	95.7%
			10%	78.0%
		Global	1%	96.7%
			5%	52.8%
			10%	11.8%
	Spectral	Local	1%	98.5%
			5%	89.1%
			10%	69.9%
		Global	1%	90.3%
			5%	46.2%
			10%	18.0%

Search Properties

AB SCIEX

General

Group filename	2jSET11.group
Start date	22-01-19
Start time	01/22/2019 11:34:00
User name	PC26635\Sabine

Search Input

Number of spectra in search	88035
Number of input files	1

Search Method

Sample Type	Identification
Cysteine Alkylation	Iodoacetamide
Digestion	Trypsin
Special Factors	
Instrument	TripleTOF 5600
Species	
Search Effort	Thorough
ID Focus	Biological modifications; Amino acid substitutions
FDR Analysis	Yes
Quantitation	
Background Correction	No
Bias Correction	Yes
Channel to use as denominator in ratios	
Modified Data Dictionary or Parameter Translation	Yes

Database Searched

Database filename	C:\AB SCIEX\ProteinPilot Data\SearchDatabases\2JSET11_2rd_DBs.fasta
Number of Proteins in Database	15254
Number of proteins searched	30508

Hardware and Software Environment

RAM (MB)	65458
Computer name	PC26635
Number of cores in computer	10
Number of threads (double cores if hyperthreading on)	20
Number of cores licensed to use	8
Operating system	Windows 7 Professional Service Pack 1 (6.1.7601)
ProteinPilot Software Version	5.0.1.0, 4895
Paragon Algorithm Version	5.0.1.0, 4874

Sample name: 2nSET12 - Metaproteome name: free-living night 1

ProteinPilot™ Software Report

Template Version 1.00 light report

Summary of Identification Yields

	Data Level	FDR Type	FDR	ID Yield
Identification Yield at FDR Threshold	Protein	Local	1%	392
			5%	437
			10%	461
		Global	1%	473
			5%	587
			10%	740
	Distinct peptide	Local	1%	750
			5%	1190
			10%	1630
		Global	1%	1147
			5%	2095
			10%	3177
	Spectral	Local	1%	3723
			5%	6607
			10%	8348
		Global	1%	6033
			5%	10401
			10%	12742
Corresponding Confidence in ProteinPilot™ Software	Protein	Local	1%	93.1%
			5%	87.4%
			10%	83.0%
		Global	1%	79.6%
			5%	48.7%
			10%	16.8%
	Distinct peptide	Local	1%	99.1%
			5%	94.9%
			10%	81.1%
		Global	1%	95.6%
			5%	54.7%
			10%	14.3%
	Spectral	Local	1%	98.6%
			5%	87.1%
			10%	69.1%
		Global	1%	90.7%
			5%	46.4%
			10%	15.5%

Search Properties

AB SCIEX

General

Group filename	2nSET12.group
Start date	22-01-19
Start time	01/22/2019 11:35:16
User name	PC26635\Sabine

Search Input

Number of spectra in search	82451
Number of input files	1

Search Method

Sample Type	Identification
Cysteine Alkylation	Iodoacetamide
Digestion	Trypsin
Special Factors	
Instrument	TripleTOF 5600
Species	
Search Effort	Thorough
ID Focus	Biological modifications; Amino acid substitutions
FDR Analysis	Yes
Quantitation	
Background Correction	No
Bias Correction	Yes
Channel to use as denominator in ratios	
Modified Data Dictionary or Parameter Translation	Yes

Database Searched

Database filename	C:\AB SCIEX\ProteinPilot Data\SearchDatabases\2nSET12_2rd_DBs.fasta
Number of Proteins in Database	13774
Number of proteins searched	27548

Hardware and Software Environment

RAM (MB)	65458
Computer name	PC26635
Number of cores in computer	10
Number of threads (double cores if hyperthreading on)	20
Number of cores licensed to use	8
Operating system	Windows 7 Professional Service Pack 1 (6.1.7601)
ProteinPilot Software Version	5.0.1.0, 4895
Paragon Algorithm Version	5.0.1.0, 4874

Sample name: 2j2SET13 - Metaproteome name: free-living day 2

ProteinPilot™ Software Report

Template Version 1.00 light report

Summary of Identification Yields

	Data Level	FDR Type	FDR	ID Yield
Identification Yield at FDR Threshold	Protein	Local	1%	505
			5%	507
			10%	550
		Global	1%	568
			5%	720
			10%	846
	Distinct peptide	Local	1%	1048
			5%	1181
			10%	1775
		Global	1%	1328
			5%	2342
			10%	3954
	Spectral	Local	1%	3884
			5%	6060
			10%	7610
		Global	1%	5903
			5%	9616
			10%	11818
Corresponding Confidence in ProteinPilot™ Software	Protein	Local	1%	90.7%
			5%	90.5%
			10%	83.0%
		Global	1%	78.1%
			5%	36.9%
			10%	14.9%
	Distinct peptide	Local	1%	98.4%
			5%	97.1%
			10%	81.6%
		Global	1%	95.4%
			5%	46.4%
			10%	0.1%
	Spectral	Local	1%	98.9%
			5%	91.6%
			10%	77.5%
		Global	1%	92.8%
			5%	50.8%
			10%	15.5%

Search Properties

General

Group filename	2j2SET13.group
Start date	22-01-19
Start time	01/22/2019 11:33:21
User name	PC26635\Sabine

Search Input

Number of spectra in search	75910
Number of input files	1

Search Method

Sample Type	Identification
Cysteine Alkylation	Iodoacetamide
Digestion	Trypsin
Special Factors	
Instrument	TripleTOF 5600
Species	
Search Effort	Thorough
ID Focus	Biological modifications; Amino acid substitutions
FDR Analysis	Yes
Quantitation	
Background Correction	No
Bias Correction	Yes
Channel to use as denominator in ratios	
Modified Data Dictionary or Parameter Translation	Yes

Database Searched

Database filename	C:\AB SCIEX\ProteinPilot Data\SearchDatabases\2J2SET13_2rd_DBs.fasta
Number of Proteins in Database	18504
Number of proteins searched	37008

Hardware and Software Environment

RAM (MB)	65458
Computer name	PC26635
Number of cores in computer	10
Number of threads (double cores if hyperthreading on)	20
Number of cores licensed to use	8
Operating system	Windows 7 Professional Service Pack 1 (6.1.7601)
ProteinPilot Software Version	5.0.1.0, 4895
Paragon Algorithm Version	5.0.1.0, 4874

Sample name: 2n2SET14 - Metaproteome name: free-living night 2

ProteinPilot™ Software Report

Template Version 1.00 light report

Summary of Identification Yields

	Data Level	FDR Type	FDR	ID Yield
Identification Yield at FDR Threshold	Protein	Local	1%	411
			5%	452
			10%	473
		Global	1%	485
			5%	592
			10%	772
	Distinct peptide	Local	1%	707
			5%	1131
			10%	1570
		Global	1%	1082
			5%	2012
			10%	3124
	Spectral	Local	1%	4234
			5%	6353
			10%	7827
		Global	1%	6331
			5%	9918
			10%	12121
Corresponding Confidence in ProteinPilot™ Software	Protein	Local	1%	95.9%
			5%	91.5%
			10%	88.0%
		Global	1%	85.9%
			5%	55.3%
			10%	18.7%
	Distinct peptide	Local	1%	99.6%
			5%	96.2%
			10%	80.0%
		Global	1%	97.0%
			5%	54.3%
			10%	12.9%
	Spectral	Local	1%	98.6%
			5%	87.5%
			10%	68.4%
		Global	1%	87.9%
			5%	40.4%
			10%	17.4%

Search Properties

AB SCIEX

General

Group filename	2n2SET14.group
Start date	22-01-19
Start time	01/22/2019 11:34:41
User name	PC26635\Sabine

Search Input

Number of spectra in search	72101
Number of input files	1

Search Method

Sample Type	Identification
Cysteine Alkylation	Iodoacetamide
Digestion	Trypsin
Special Factors	
Instrument	TripleTOF 5600
Species	
Search Effort	Thorough
ID Focus	Biological modifications; Amino acid substitutions
FDR Analysis	Yes
Quantitation	
Background Correction	No
Bias Correction	Yes
Channel to use as denominator in ratios	
Modified Data Dictionary or Parameter Translation	Yes

Database Searched

Database filename	C:\AB SCIEX\ProteinPilot Data\SearchDatabases\2n2SET14_2rd_DBs.fasta
Number of Proteins in Database	15438
Number of proteins searched	30876

Hardware and Software Environment

RAM (MB)	65458
Computer name	PC26635
Number of cores in computer	10
Number of threads (double cores if hyperthreading on)	20
Number of cores licensed to use	8
Operating system	Windows 7 Professional Service Pack 1 (6.1.7601)
ProteinPilot Software Version	5.0.1.0, 4895
Paragon Algorithm Version	5.0.1.0, 4874

Sample name: 8jSET18 - Metaproteome name: particle-attached day 1

ProteinPilot™ Software Report

Template Version 1.00 light report

Summary of Identification Yields

	Data Level	FDR Type	FDR	ID Yield
Identification Yield at FDR Threshold	Protein	Local	1%	0
			5%	0
			10%	0
		Global	1%	161
			5%	161
			10%	161
	Distinct peptide	Local	1%	680
			5%	680
			10%	680
		Global	1%	1160
			5%	1160
			10%	1160
	Spectral	Local	1%	390
			5%	624
			10%	797
		Global	1%	601
			5%	1007
			10%	1278
Corresponding Confidence in ProteinPilot™ Software	Protein	Local	1%	
			5%	
			10%	
		Global	1%	10.9%
			5%	10.9%
			10%	10.9%
	Distinct peptide	Local	1%	12.4%
			5%	12.4%
			10%	12.4%
		Global	1%	0.1%
			5%	0.1%
			10%	0.1%
	Spectral	Local	1%	99.9%
			5%	94.0%
			10%	81.1%
		Global	1%	95.4%
			5%	61.8%
			10%	34.5%

Search Properties

General

Group filename	8jSET18(1).group
Start date	09-09-21
Start time	16:03:14
User name	Bioprofil4\UMons

Search Input

Number of spectra in search	88177
Number of input files	1

Search Method

Sample Type	Identification
Cysteine Alkylation	Iodoacetamide
Digestion	Trypsin
Special Factors	
Instrument	TripleTOF 5600
Species	
Search Effort	Thorough
ID Focus	Biological modifications; Amino acid substitutions
FDR Analysis	No
Quantitation	
Background Correction	No
Bias Correction	No
Channel to use as denominator in ratios	
Modified Data Dictionary or Parameter Translation	No

Database Searched

Database filename	C:\AB SCIEX\ProteinPilot Data\SearchDatabases\08_Second_round_DB.fasta
Number of Proteins in Database	20038
Number of proteins searched	20038

Hardware and Software Environment

RAM (MB)	98225
Computer name	BIOPROFIL4
Number of cores in computer	16
Number of threads (double cores if hyperthreading on)	32
Number of cores licensed to use	16
Operating system	Windows 7 Professional Service Pack 1 (6.1.7601)
ProteinPilot Software Version	5.0.1.0, 4895
Paragon Algorithm Version	5.0.1.0, 4874

Sample name: 8nSET19 - Metaproteome name: particle-attached night 1

ProteinPilot™ Software Report

Template Version 1.00 light report

Summary of Identification Yields

	Data Level	FDR Type	FDR	ID Yield
Identification Yield at FDR Threshold	Protein	Local	1%	0
			5%	0
			10%	0
		Global	1%	182
			5%	182
			10%	182
	Distinct peptide	Local	1%	670
			5%	670
			10%	670
		Global	1%	1219
			5%	1219
			10%	1219
	Spectral	Local	1%	1142
			5%	1662
			10%	1874
		Global	1%	1619
			5%	2275
			10%	2603
Corresponding Confidence in ProteinPilot™ Software	Protein	Local	1%	
			5%	
			10%	
		Global	1%	10.9%
			5%	10.9%
			10%	10.9%
	Distinct peptide	Local	1%	21.1%
			5%	21.1%
			10%	21.1%
		Global	1%	0.1%
			5%	0.1%
			10%	0.1%
	Spectral	Local	1%	99.7%
			5%	85.9%
			10%	70.2%
		Global	1%	89.3%
			5%	35.3%
			10%	18.0%

Search Properties

General

Group filename	8nSET19.group
Start date	09-09-21
Start time	16:03:42
User name	Bioprofil4\UMons

Search Input

Number of spectra in search	87643
Number of input files	1

Search Method

Sample Type	Identification
Cysteine Alkylation	Iodoacetamide
Digestion	Trypsin
Special Factors	
Instrument	TripleTOF 5600
Species	
Search Effort	Thorough
ID Focus	Biological modifications; Amino acid substitutions
FDR Analysis	No
Quantitation	
Background Correction	No
Bias Correction	No
Channel to use as denominator in ratios	
Modified Data Dictionary or Parameter Translation	No

Database Searched

Database filename	C:\AB SCIEX\ProteinPilot Data\SearchDatabases\08_Second_round_DB.fasta
Number of Proteins in Database	20038
Number of proteins searched	20038

Hardware and Software Environment

RAM (MB)	98225
Computer name	BIOPROFIL4
Number of cores in computer	16
Number of threads (double cores if hyperthreading on)	32
Number of cores licensed to use	16
Operating system	Windows 7 Professional Service Pack 1 (6.1.7601)
ProteinPilot Software Version	5.0.1.0, 4895
Paragon Algorithm Version	5.0.1.0, 4874

Sample name: 8j2SET110 - Metaproteome name: particle-attached day 2

ProteinPilot™ Software Report

Template Version 1.00 light report

Summary of Identification Yields

	Data Level	FDR Type	FDR	ID Yield
Identification Yield at FDR Threshold	Protein	Local	1%	0
			5%	0
			10%	0
		Global	1%	197
			5%	197
			10%	197
	Distinct peptide	Local	1%	1455
			5%	1455
			10%	1455
		Global	1%	1681
			5%	1681
			10%	1681
	Spectral	Local	1%	1580
			5%	2197
			10%	2476
		Global	1%	2262
			5%	3049
			10%	3488
Corresponding Confidence in ProteinPilot™ Software	Protein	Local	1%	
			5%	
			10%	
		Global	1%	10.9%
			5%	10.9%
			10%	10.9%
	Distinct peptide	Local	1%	4.0%
			5%	4.0%
			10%	4.0%
		Global	1%	0.1%
			5%	0.1%
			10%	0.1%
	Spectral	Local	1%	99.4%
			5%	86.1%
			10%	72.6%
		Global	1%	84.6%
			5%	37.6%
			10%	16.5%

Search Properties

AB SCIEX

General

Group filename	8j2SET110.group
Start date	09-09-21
Start time	16:03:28
User name	Bioprofil4\UMons

Search Input

Number of spectra in search	86844
Number of input files	1

Search Method

Sample Type	Identification
Cysteine Alkylation	Iodoacetamide
Digestion	Trypsin
Special Factors	
Instrument	TripleTOF 5600
Species	
Search Effort	Thorough
ID Focus	Biological modifications; Amino acid substitutions
FDR Analysis	No
Quantitation	
Background Correction	No
Bias Correction	No
Channel to use as denominator in ratios	
Modified Data Dictionary or Parameter Translation	No

Database Searched

Database filename	C:\AB SCIEX\ProteinPilot Data\SearchDatabases\08_Second_round_DB.fasta
Number of Proteins in Database	20038
Number of proteins searched	20038

Hardware and Software Environment

RAM (MB)	98225
Computer name	BIOPROFIL4
Number of cores in computer	16
Number of threads (double cores if hyperthreading on)	32
Number of cores licensed to use	16
Operating system	Windows 7 Professional Service Pack 1 (6.1.7601)
ProteinPilot Software Version	5.0.1.0, 4895
Paragon Algorithm Version	5.0.1.0, 4874

Sample name: 8n2SET111 - Metaproteome name: particle-attached night 2

ProteinPilot™ Software Report

Template Version 1.00 light report

Summary of Identification Yields

	Data Level	FDR Type	FDR	ID Yield
Identification Yield at FDR Threshold	Protein	Local	1%	190
			5%	190
			10%	192
		Global	1%	225
			5%	235
			10%	235
	Distinct peptide	Local	1%	639
			5%	639
			10%	639
		Global	1%	1858
			5%	1858
			10%	1858
	Spectral	Local	1%	1977
			5%	2693
			10%	3016
		Global	1%	2813
			5%	3705
			10%	4255
Corresponding Confidence in ProteinPilot™ Software	Protein	Local	1%	59.3%
			5%	59.3%
			10%	53.2%
		Global	1%	14.9%
			5%	10.9%
			10%	10.9%
	Distinct peptide	Local	1%	86.8%
			5%	86.8%
			10%	86.8%
		Global	1%	0.1%
			5%	0.1%
			10%	0.1%
	Spectral	Local	1%	99.2%
			5%	88.8%
			10%	74.1%
		Global	1%	82.9%
			5%	32.2%
			10%	16.9%

Search Properties

General

Group filename	8n2SET111.group
Start date	09-09-21
Start time	16:03:57
User name	Bioprofil4\UMons

Search Input

Number of spectra in search	86537
Number of input files	1

Search Method

Sample Type	Identification
Cysteine Alkylation	Iodoacetamide
Digestion	Trypsin
Special Factors	
Instrument	TripleTOF 5600
Species	
Search Effort	Thorough
ID Focus	Biological modifications; Amino acid substitutions
FDR Analysis	No
Quantitation	
Background Correction	No
Bias Correction	No
Channel to use as denominator in ratios	
Modified Data Dictionary or Parameter Translation	No

Database Searched

Database filename	C:\AB SCIEX\ProteinPilot Data\SearchDatabases\08_Second_round_DB.fasta
Number of Proteins in Database	20038
Number of proteins searched	20038

Hardware and Software Environment

RAM (MB)	98225
Computer name	BIOPROFIL4
Number of cores in computer	16
Number of threads (double cores if hyperthreading on)	32
Number of cores licensed to use	16
Operating system	Windows 7 Professional Service Pack 1 (6.1.7601)
ProteinPilot Software Version	5.0.1.0, 4895
Paragon Algorithm Version	5.0.1.0, 4874
